# Heterogeneity in Treatment Effects of Reduced Versus Standard Dose of Cabazitaxel in Metastatic Castration‐Resistant Prostate Cancer

**DOI:** 10.1002/cam4.71507

**Published:** 2026-01-09

**Authors:** Wataru Fukuokaya, Keiichiro Mori, Takafumi Yanagisawa, Kagenori Ito, Fumihiko Urabe, Pawel Rajwa, Shahrokh F. Shariat, Takahiro Kimura, Akihiro Hirakawa

**Affiliations:** ^1^ Department of Clinical Biostatistics Institute of Science Tokyo Tokyo Japan; ^2^ Department of Urology The Jikei University School of Medicine Tokyo Japan; ^3^ Department of Urology Medical University of Silesia Zabrze Poland; ^4^ Department of Urology, Comprehensive Cancer Center Medical University of Vienna Vienna Austria; ^5^ Division of Surgery and Interventional Science University College London London UK; ^6^ Department of Urology University of Texas Southwestern Medical Center Dallas Texas USA; ^7^ Department of Urology, Second Faculty of Medicine Charles University Prague Czech Republic; ^8^ Department of Urology Weill Cornell Medical College New York New York USA; ^9^ Karl Landsteiner Institute of Urology and Andrology Vienna Austria

**Keywords:** cabazitaxel, effect‐based modeling, heterogeneous treatment effect, metastatic castration‐resistant prostate cancer, risk‐based modeling

## Abstract

**Background:**

In the PROSELICA, a randomized controlled trial (RCT) comparing cabazitaxel 20 mg/m^2^ (C20) versus 25 mg/m^2^ (C25) in metastatic castration‐resistant prostate cancer (mCRPC), one‐variable‐at‐a‐time subgroup analysis suggested possible heterogeneity in treatment effect (HTE) of C25 versus C20 among study participants. Novel predictive HTE analysis approaches may provide an in‐depth understanding of such results.

**Methods:**

We analyzed patient‐level data from 1200 patients with mCRPC who were randomized in the PROSELICA trial. Outcomes included overall survival (OS) and progression‐free survival (PFS). Using baseline characteristics, patients were stratified into quartiles based on either quantitative baseline risk of poor outcome (risk modeling) or predicted individualized treatment effect (ITE) using a causal survival forest algorithm (effect modeling). Treatment effects were measured as differences in restricted mean survival time (RMST).

**Results:**

For risk modeling, the OS effect of C25 increased with risk quartiles: −0.07 months (95% CI, −1.60 to 1.46) in the lowest risk quartile and 1.67 months (95% CI, 0.25 to 3.10) in the highest risk quartile. For effect modeling, the OS effect ranged from −0.17 months (95% CI, −3.01 to 2.68) in the lowest ITE quartile to 0.57 months (95% CI, −2.27 to 3.41) in the highest ITE quartile. Both approaches demonstrated greater C25 benefit in patients with extensive previous treatment and baseline disease burden. PFS effects remained consistent across all quartiles.

**Conclusions:**

The OS effect of C25 versus C20 may vary based on baseline characteristics in post‐docetaxel mCRPC. Patients with extensive treatment history and disease burden may benefit more from C25.

## 1 Introduction

Metastatic castration‐resistant prostate cancer (mCRPC) is a lethal disease with a median survival of approximately 2 years from mCRPC diagnosis [1]. Among available treatment options, cabazitaxel 25 mg/m^2^ (C25) plus prednisone demonstrated efficacy over mitoxantrone in patients with mCRPC who were previously treated with docetaxel, based on results of the TROPIC trial [2]. Data also showed a high incidence of severe adverse events with cabazitaxel. The PROSELICA trial, a post‐marketing requirement, aimed to demonstrate non‐inferiority of cabazitaxel 20 mg/m^2^ (C20) versus C25 in patients with mCRPC who experienced disease progression during or after docetaxel [3]. This trial demonstrated non‐inferiority of C20 versus C25 for overall survival (OS), with a lower incidence of treatment‐related adverse events of grade 3 or higher for C20 (39.7% vs. 54.5%).

The subgroup analysis for OS in this trial suggested possible heterogeneity in treatment effect (HTE) of C25 over C20 for baseline characteristics such as prior abiraterone acetate use, tumor burden, and lactate dehydrogenase levels [3]. However, such one‐variable‐at‐a‐time subgroup analysis has substantial limitations, including multiple comparisons resulting in false‐positive findings and poor statistical power resulting in false‐negative findings [4, 5, 6, 7]. To address these limitations, rapid advancements have been achieved in predictive HTE analysis using a multivariable risk‐modeling approach and an effect‐modeling approach with machine learning algorithms [8, 9, 10, 11]. Using these two newer approaches could provide more valid estimates for the possible HTE of C25 over C20 in this population, potentially optimizing the use of cabazitaxel, which would be important given the limited active treatment options available for post‐docetaxel mCRPC.

In this exploratory post hoc analysis, we conducted these two novel predictive HTE analyses to investigate possible HTE of C25 versus C20 based on data from two randomized controlled trials (RCTs) of cabazitaxel treatment in patients with mCRPC who were previously treated with docetaxel.

## 2 Methods

This study follows the Strengthening the Reporting of Observational Studies in Epidemiology Statement [12] and the Predictive Approaches to treatment effect Heterogeneity statement [11].

### 2.1 Study Design and Population

This study analyzed patient‐level data from the PROSELICA trial and the TROPIC trial. The PROSELICA trial was a phase III RCT designed to test the non‐inferiority of C20 versus C25, both administered with prednisone 10 mg and androgen deprivation therapy (ADT), in patients with mCRPC who developed disease progression during or after docetaxel (ClinicalTrials.gov number, NCT01308580). In this trial, 1200 patients were randomized 1:1 to either C20 (*N* = 598) or C25 (*N* = 602) between April 2011 and December 2013 [3]. The detailed eligibility criteria and results of this trial were previously reported elsewhere [3].

The TROPIC trial was a phase III RCT comparing C25 and mitoxantrone in patients with mCRPC who experienced disease progression during or after docetaxel [2]. In this trial, 755 patients were randomly allocated at a 1:1 ratio to either cabazitaxel (*N* = 378) or mitoxantrone (*N* = 377). We used the TROPIC data for external validation of the risk models. Therefore, only data from the cabazitaxel arm were analyzed. The details of the risk model analysis are described in the Statistical Analysis section.

### 2.2 Outcomes

The outcomes of interest were OS, measured as the time from randomization to death from any cause, and progression‐free survival (PFS), defined as the time from randomization to the first documentation of radiological progression, prostate‐specific antigen (PSA) progression, pain progression, or death from any cause [2, 3]. The incidence of grade ≥ 3 non‐hematological treatment‐related adverse events (trAEs), grade ≥ 3 neutropenia, and febrile neutropenia was also evaluated, with trAEs graded using the National Cancer Institute Common Terminology Criteria for Adverse Events version 4.03.

### 2.3 Statistical Analysis

HTE was evaluated using the risk‐modeling and effect‐modeling approaches. In both approaches, the effect of C25 versus C20 was quantified as the difference in 24‐month restricted mean survival time (RMST) for OS and difference in 6‐month RMST for PFS to provide clinically meaningful measures for absolute treatment effect [13]. Difference in RMST is a mean survival time difference up to a truncated timepoint. A difference in RMST > 0 indicated better survival with C25 than with C20.

#### 2.3.1 Risk‐Modeling Approach

This approach examined HTE across levels of risk for poor outcomes. The risk‐modeling analysis uses a multivariable predictive model that stratifies patients according to their calculated risk scores to examine how treatment effects vary based on these predicted levels.

To quantify risk for each patient, multivariable Cox proportional hazards regression models for OS and PFS were developed using the intention‐to‐treat population from the PROSELICA trial and 17 baseline characteristics presented in Table 1. Details about the data types of candidate variables are provided in the Supporting Information. Among all possible combinations of candidate variables (*N* = 2^17^), the model with the lowest Akaike Information Criterion was selected. To ensure that each patient's risk score reflected their baseline prognosis independent of treatment assignment, the final risk model for each outcome was adjusted for treatment allocation. The quantitative risk for each patient was then computed from the model's coefficients, assuming all patients were in the control arm.

**TABLE 1:** Baseline characteristics.

Characteristic	C20	C25
	*N* = 598	*N* = 602
Age at baseline, *n* (%)		
Less than 60	86 (14.4)	89 (14.8)
60 to 80	486 (81.3)	482 (80.1)
More than 80	26 (4.3)	31 (5.1)
Body mass index, *n* (%)		
Underweight to normal weight	175 (29.3)	175 (29.1)
Pre‐obesity	263 (44.0)	282 (46.9)
Obesity class I or more	160 (26.8)	144 (24.0)
Missing	0	1
ECOG performance status, *n* (%)		
0	211 (35.3)	171 (28.4)
1 or more	387 (64.7)	431 (71.6)
Present pain intensity, median (IQR)	1.0 (0.0 to 2.0)	2.0 (0.0 to 2.0)
Missing	56	41
Baseline opioid use, *n* (%)	239 (40.0)	267 (44.4)
Prior androgen receptor pathway inhibitor, *n* (%)	148 (24.7)	160 (26.6)
Baseline disease activity, *n* (%)		
Increasing PSA at baseline	526 (88.0)	518 (86.0)
Stable or declining PSA at baseline	72 (12.0)	84 (14.0)
Time from last docetaxel dose to progression, median (IQR)	1.0 (0.0 to 3.6)	1.0 (0.0 to 3.4)
Missing	53	56
Number of prior chemotherapies, *n* (%)		
1	480 (82.3)	495 (82.4)
2 or more	103 (17.7)	106 (17.6)
Missing	15	1
Time on hormonal therapy, median (IQR)	3.1 (2.0 to 5.3)	3.1 (1.8 to 5.3)
Missing	98	98
Baseline bone metastasis, *n* (%)		
Abscent	39 (6.5)	33 (5.5)
Present	559 (93.5)	569 (94.5)
Baseline lymph node metastasis, *n* (%)		
Abscent	304 (50.8)	303 (50.3)
Present	294 (49.2)	299 (49.7)
Baseline visceral metastasis, *n* (%)		
Abscent	430 (71.9)	435 (72.3)
Present	168 (28.1)	167 (27.7)
Baseline hemoglobin concentration, median (IQR)	119.1 (107.9 to 130.0)	120.0 (109.5 to 129.0)
Baseline NLR, median (IQR)	3.4 (2.2 to 5.4)	3.4 (2.3 to 5.5)
Missing	4	4
Baseline ALP, median (IQR)	166.0 (91.0 to 357.0)	166.5 (96.0 to 337.0)
Missing	1	4
Baseline PSA, median (IQR)	159.5 (51.0 to 431.3)	170.9 (59.9 to 429.0)
Missing	4	5

Abbreviations: ALP, alkaline phosphatase; IQR, interquartile range; NLR, neutrophil‐to‐lymphocyte ratio; PSA, prostate‐specific antigen.

The risk scores were evaluated as a potential effect modifier by computing the conditional average treatment effect—the treatment effect within patient subgroups defined by a shared set of characteristics—within each risk quartile. Reasons for discontinuation and the incidence of severe trAEs, neutropenia, and febrile neutropenia were compared by risk quartiles. For the analysis of trAEs, the safety analysis dataset (*N* = 1175) was used. Model discriminatory performance was evaluated using Harrell's c‐statistic on data from the PROSELICA trial and the C25 arm of the TROPIC trial. Model calibration was assessed visually using calibration plots [14].

#### 2.3.2 Effect‐Modeling Approach

The effect‐modeling approach develops a model based directly on data to predict treatment effects. Treatment effects were evaluated across levels of predicted individualized treatment effect (ITE), estimated using a causal survival forest algorithm based on machine learning [15]. This algorithm is an extension of the random forest algorithm; while the random forest optimizes predictions of outcome risks, the causal survival forest optimizes the prediction of differences in outcome risks between treatment groups [16]. We applied the generic machine learning framework for inference on HTEs proposed by Chernozukhov et al. [17] In this framework, Monte‐Carlo cross‐validation with 100 repetitions was used to estimate cATEs within ITE quartiles, with 95% confidence intervals (CIs). The contributions of each variable to the treatment effect predictions were quantified using variable importance. Patient characteristics were also compared by ITE quartiles.

Null hypothesis testing for statistical interaction was not performed for the effect of C25 on risk or on ITE quartiles, as non‐significant results likely reflect low power rather than a true lack of effect modification on the risk difference scale [13].

Detailed statistical analyses are provided in Supporting Information and were performed using R version 4.3.1 (R Foundation For Statistical Computing).

## 3 Results

### 3.1 Baseline Characteristics of the PROSELICA Trial

The median follow‐up time was 33.8 months (interquartile range [IQR], 27.2 to 39.8). During follow‐up, 88.4% of patients (1061 of 1200) experienced disease progression, and 83.2% (998 of 1200) died. For OS, the 24‐month RMSTs were 14.0 months (95% CI, 13.3 to 14.6) for C20 and 14.5 months (95% CI, 13.9 to 15.1) for C25, with a difference of 0.5 months (95% CI, −0.3 to 1.4) (Figure S1). For PFS, the 6‐month RMSTs were 3.3 months (95% CI, 3.1 to 3.5) for C20 and 3.5 months (95% CI, 3.3 to 3.7) for C25, with a difference of 0.2 months (95% CI, 0.01 to 0.5) (Figure S1). At baseline, 25.7% of patients (308 of 1200) had prior ARPI use and 27.9% (335 of 1200) had visceral metastasis (Table 1). The median time from last docetaxel dose to progression was 1.0 months (95% CI, 0.0 to 3.5), and the median time on hormonal therapy was 3.1 years (95% CI, 1.9 to 5.3).

### 3.2 Baseline Characteristics of the TROPIC Trial

For patients randomized to C25, the median follow‐up time was 20.7 months (IQR, 15.1 to 24.5). During follow‐up, 96.3% of patients (364 of 378) experienced disease progression, and 61.9% (234 of 378) died. The 24‐month RMST for OS was 15.2 months (95% CI, 14.4 to 16.0), and the 6‐month RMST for PFS was 3.2 months (95% CI, 3.0 to 3.4) (Figure S1). At baseline, none of the TROPIC population had prior ARPI use, and 26.7% (101 of 378) had visceral metastasis (Table S1). The median time from last docetaxel dose to progression was 0.8 months (95% CI, 0.0 to 3.4), while the median time on hormonal therapy was 4.2 years (95% CI, 2.5 to 6.6).

### 3.3 Risk‐Modeling Approach for Heterogeneity in Treatment Effect

The final risk models for OS and PFS are detailed in Table 2. In the PROSELICA population, the distribution of risk scores for both outcomes was observed to be largely symmetric and unimodal (Figure S2). Higher risk scores had higher HR for OS (HR, 2.72; 95% CI, 2.44 to 3.03) and PFS (HR, 2.72; 95% CI, 2.19 to 3.37). The c‐statistic for the OS risk model was 0.65 in the PROSELICA dataset and 0.72 in the TROPIC dataset, while for the PFS risk model, it was 0.55 and 0.54, respectively. Calibration plots showed that the risk models for OS and PFS were well‐calibrated in both the PROSELICA and the TROPIC datasets (Figure S3).

**TABLE 2:** Risk model used for risk‐modeling heterogeneity of treatment effect analysis.

Characteristic	HR (95% CI)	Log (HR)
Overall survival risk model		
ECOG performance status (0 vs. ≥ 1)	1.33 (1.15 to 1.53)	0.28
Prior ARPI (no vs. yes)	1.25 (1.08 to 1.44)	0.22
Present pain intensity (continuous)	1.12 (1.05 to 1.19)	0.11
Baseline opioid use (no vs. yes)	1.16 (1.01 to 1.34)	0.15
Bone metastasis (absent vs. present)	1.61 (1.18 to 2.20)	0.48
Lymph node metastasis (absent vs. present)	1.34 (1.18 to 1.52)	0.29
Visceral metastasis (absent vs. present)	1.31 (1.14 to 1.51)	0.27
Time from last docetaxel dose to progression, month (continuous)	0.98 (0.97 to 0.99)	−0.02
Time on hormonal therapy, year (continuous)	0.93 (0.90 to 0.95)	−0.08
Baseline hemoglobin, per 10 g/L (continuous)	0.83 (0.79 to 0.87)	−0.19
Baseline NLR, per 10 (continuous)	1.01 (1.00 to 1.02)	0.01
Baseline ALP, per 100 IU/L (continuous)	1.02 (1.02 to 1.03)	0.02
Progression‐free survival risk model		
Present pain intensity (continuous)	1.17 (1.10 to 1.24)	0.16
Baseline opioid use (no vs. yes)	1.11 (0.97 to 1.28)	0.11
Visceral metastasis (absent vs. present)	1.13 (0.98 to 1.29)	0.12
Baseline disease activity (stable or declining PSA vs. increasing PSA)	0.85 (0.71 to 1.02)	−0.16
Time from last docetaxel dose to progression, month (continuous)	0.99 (0.98 to 1.00)	−0.01
Time on hormonal therapy, year (continuous)	0.95 (0.93 to 0.97)	−0.05
Number of prior chemotherapies (1 or ≥ 2)	1.15 (0.98 to 1.36)	0.14
Baseline hemoglobin, per 10 g/L (continuous)	0.95 (0.91 to 0.99)	−0.05

**FIGURE 1:**
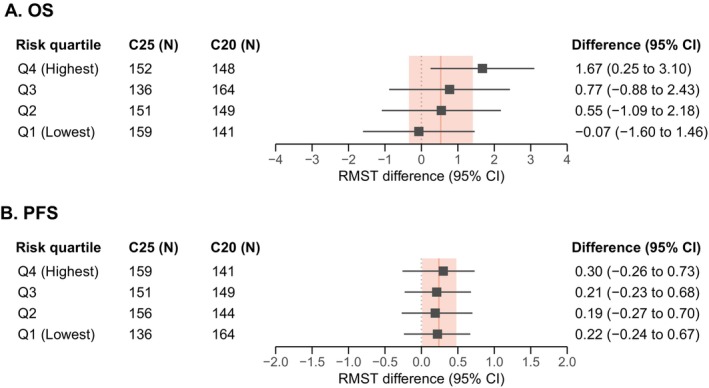
Restricted mean survival time difference based on risk quartiles. Forest plots showing restricted mean survival time (RMST) difference by risk quartiles, ranging from the lowest risk quartile (Q1) to the highest risk quartile (Q4). The truncation point was set at 24 months for OS and 6 months for PFS. RMST difference of > 0 indicates that patients receiving cabazitaxel 25 mg/m^2^ (C25) had better survival than those treated with cabazitaxel 20 mg/m^2^ (C20). The red, solid line indicates the average treatment effect of C25 versus C20, with red bands showing 95% confidence intervals, in the intention‐to‐treat population of the PROSELICA trial (average treatment effect for OS, 0.5 months [95% CI, −0.3 to 1.4]; average treatment effect for PFS, 0.25 months [95% CI, 0.02 to 0.48]).

The effect of C25 over C20 by risk quartile is shown in Figure 1. For OS, observed RMST differences increased with risk quartiles. RMST difference was 1.67 months (95% CI, 0.25 to 3.10) in the highest risk quartile and −0.07 months (−1.60 to 1.46) in the lowest risk quartile. In contrast, no such association was observed for PFS, where the RMST difference was similar between the highest (0.30 months; 95% CI, −0.26 to 0.73) and lowest (0.22 months; 95% CI, −0.24 to 0.67) risk quartiles.

**FIGURE 2:**
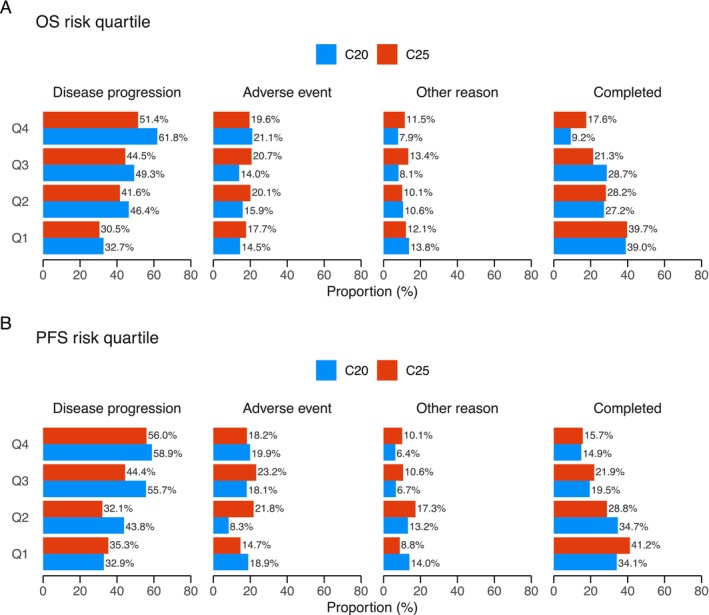
Reasons for treatment discontinuation based on risk quartiles. Bar charts showing reasons for treatment discontinuation based on risk quartiles. The PROSELICA trial capped cabazitaxel treatment to 10 cycles, so patients who completed all 10 cycles were categorized as Completed. The proportion of patients receiving cabazitaxel 25 mg/m^2^ (C25) is indicated in red, and those treated with 20 mg/m^2^ (C20) are shown in blue. Patients were ranked and grouped equally for baseline risk of poor OS or PFS. Q1 represents the lowest‐risk group, and Q4 represents the highest‐risk group.

The incidence of key grade ≥ 3 trAEs by risk quartile is shown in Figure S4. The incidence of these events was consistently higher for C25 than for C20 across all risk quartiles. Notably, for both outcomes, the incidence of trAEs increased with higher risk quartiles. Reasons for treatment discontinuation also varied by risk quartile (Figure 2). For both outcomes, the proportion of patients discontinuing treatment due to disease progression increased with higher risk quartiles, while discontinuation due to adverse events remained similar across all quartiles.

### 3.4 Effect‐Modeling Approach for Heterogeneity in Treatment Effect

**FIGURE 3:**
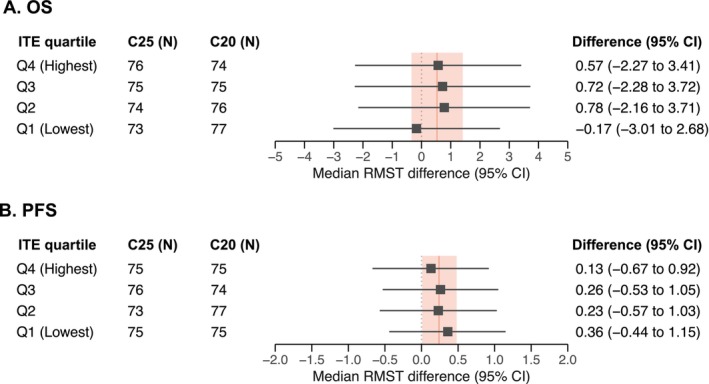
Restricted mean survival time difference based on individualized treatment effect quartiles. Forest plots showing restricted mean survival time (RMST) difference by individualized treatment effect (ITE) quartiles, ranging from the lowest ITE quartile (Q1) to the highest ITE quartile (Q4). The truncation point was set at 24 months for OS and 6 months for PFS. RMST difference of > 0 indicates that patients receiving cabazitaxel 25 mg/m^2^ had better survival than those treated with cabazitaxel 20 mg/m^2^ (C20). The solid red line indicates the average treatment effect of C25 versus C20, with red bands showing 95% confidence intervals, in the intention‐to‐treat population of the PROSELICA trial (average treatment effect for OS, 0.5 months [95% CI, −0.3 to 1.4]; average treatment effect for PFS, 0.25 months [95% CI, 0.02 to 0.48]).

The distribution of RMST differences for OS and PFS by ITE quartiles are presented in Figure 3. Patients in the lowest ITE quartile demonstrated the lowest median RMST differences for OS (−0.17 months; 95% CI, −3.01 to 2.68), while patients in the remaining ITE quartiles showed comparable RMST differences. Variable importance analysis for OS identified factors that predicted greater OS effect with C25: longer time on hormonal therapy (16.3%), higher alkaline phosphatase (14.9%), higher PSA (12.8%), higher neutrophil‐to‐lymphocyte ratio (11.5%), longer time from last docetaxel dose to progression (10.1%), and lower hemoglobin (11.8%) (Figure S4). An analysis of baseline characteristics by OS ITE quartile not only confirmed these associations but also identified additional factors associated with OS ITE, including lymph node metastasis, symptomatic disease, and an extensive pre‐treatment history (Table S2 and Figure S5). Similar associations were observed between baseline characteristics and PFS ITE quartiles (Table S3 and Figure S5).

## 4 Discussion

Although subgroup analysis of the PROSELICA trial suggested possible HTE in several subgroups, including those with elevated lactate dehydrogenase, extraskeletal metastases, or prior abiraterone use, this traditional approach has significant limitations. Analyzing variables individually increases the risk of false‐positive and false‐negative findings and fails to provide treatment effect estimates for specific patients, as multiple characteristics simultaneously influence outcomes [4, 5, 6, 7]. This predictive HTE analysis of patient‐level data from the PROSELICA and TROPIC trials yielded several key findings.

First, the OS benefit of C25 increased with a higher baseline risk of poor outcomes. Patients in the lowest ITE quartile for OS derived less benefit than those in other quartiles, suggesting possible HTE of C25. Both risk‐modeling and effect‐modeling approaches showed that more extensive pre‐treatment history, greater baseline tumor burden, and adverse blood‐based biomarkers were associated with baseline risk of poor outcome and predicted ITEs. Although the effect size across risk and ITE quartiles was modest, these findings may help optimize cabazitaxel use, given the limited effective treatments for mCRPC after docetaxel [18, 19]. However, this potential benefit must be carefully weighed against the known trAEs of C25. While the rate of treatment discontinuation due to trAEs was similar between the two dose levels, suggesting toxicities were generally manageable, our data also showed that non‐hematological trAEs increased in the highest‐risk quartiles. Recognizing that this increased non‐hematological trAEs may outweigh the OS benefit for some patients, our findings should not be interpreted as a universal recommendation for C25 in all high‐risk patients. Instead, they should be viewed as information to support a nuanced, shared decision‐making process, where a potential survival gain is balanced against an individual's risk of toxicity and treatment goals. Notably, the largely symmetric distribution of baseline risk for OS and PFS suggests that the ‘average’ patient is also the ‘typical’ patient in this cohort. Therefore, the average treatment effect reported in the trial is likely applicable to the most common type of patient within this trial population.

Second, a similar proportion of C25 and C20 patients discontinued cabazitaxel due to trAEs across risk quartiles, suggesting that the benefit‐harm balance favors patients with a higher risk of poor outcomes. Incidence of grade ≥ 3 trAEs increased with risk quartile. The increased proportion of poor baseline performance status in higher‐risk quartiles could explain the association with the risk‐associated increase in grade ≥ 3 non‐hematological trAEs, as also suggested in the previous study [20]. Of note, the PROSELICA trial avoided curative or prophylactic use of concomitant GCSF during the initial cycle, while current guidelines advise prophylactic GCSF for cabazitaxel [18]. Therefore, the association between baseline risk of poor outcomes and the incidence of trAEs may differ in clinical practice. This association should be investigated in further studies.

Third, while there was a possible association of OS risk and OS ITE with the OS effect of C25, this was less evident for PFS. The similar C25 effect for PFS across risk quartiles observed in this study may be attributed to low c‐statistic (0.54) and consistently high 6‐month outcome rates across all risk quartiles. A previous study demonstrated that treatment effect heterogeneity by risk quartile was positively associated with model c‐statistics and negatively associated with outcome prevalence [21]. A PFS risk model with improved discriminatory performance and analysis using a more heterogeneous population of patients with post‐docetaxel mCRPC, for example using real world data, might better reveal PFS‐related HTE.

Both risk‐ and effect‐modeling approaches identified patients who potentially benefited more from C25, including those with extensive baseline tumor burden and pre‐treatment history. In contrast, while the risk‐modeling approach suggested greater benefit for patients with shorter time on hormonal therapy and time from last docetaxel dose to progression, the effect‐modeling approach predicted that these patients would derive less benefit from C25. Given that both approaches identified baseline risk and that predicted ITEs were associated with increased OS effect of C25, these results suggested that baseline tumor burden and pre‐treatment history had a greater impact on the OS effect of C25 than the duration of prior treatments.

This study has several limitations. First, the biological mechanisms underlying observed HTE remain uncertain. Second, patient selection through the eligibility criteria of the PROSELICA trial may have resulted in a more homogeneous population, potentially reducing the degree of HTE among patients. Consequently, HTE might differ if cabazitaxel was administered in a real‐world setting. Third, although this study used data from the PROSELICA trial, which was the only RCT comparing C25 to C20 in post‐docetaxel mCRPC, limited statistical power for detecting HTE prevented precise effect estimates across subgroups, particularly in the effect‐modeling approach due to sample splitting. Previous studies have demonstrated that detecting a subgroup effect requires at least a four‐fold larger sample size than detecting a main effect, even under favorable conditions [6, 7]. Well‐designed, patient‐level data meta‐analyses may improve the precision of effect estimates across subgroups. Fourth, our analyses on treatment effect heterogeneity, for both the risk‐ and effect‐modeling approaches, could not be externally validated. While the prognostic performance of our risk model was assessed using the TROPIC trial data, an external validation of our findings on heterogeneity would require in further studies. Fifth, because follow‐up durations differed among the risk and effect quartiles, the truncation time was set at 24 months. Further studies are warranted to investigate the long‐term effects of C25 according to baseline risk of poor outcomes or predicted ITE. Finally, the PROSELICA trial enrolled patients between 2011 and 2013, a period before ARPIs were widely used in earlier disease states. This represents a significant difference from the current patient population, where most patients have received prior ARPIs. While the median OS in the C25 arm of PROSELICA (14.5 months) is comparable to that in the more contemporary CARD trial [22] (13.6 months), the distribution of prognostic factors might possibly change. Therefore, the generalizability of our findings to the modern treatment landscape requires careful consideration.

## 5 Conclusions

This predictive HTE analysis suggested that the effect of C25 versus C20 may vary based on baseline characteristics in post‐docetaxel mCRPC. The effect was larger for those with more extensive pre‐treatment history and tumor burden. The proportion of patients who discontinued cabazitaxel treatment due to trAEs was similar across baseline risk of poor outcomes.

## Author Contributions

**Wataru Fukuokaya:** conceptualization (equal), data curation (equal), formal analysis (equal), methodology (equal), writing – original draft (equal). **Keiichiro Mori:** conceptualization (equal), data curation (equal), writing – review and editing (equal). **Takafumi Yanagisawa:** data curation (equal), writing – review and editing (equal). **Kagenori Ito:** data curation (equal), writing – original draft (equal). **Fumihiko Urabe:** writing – original draft (equal). **Pawel Rajwa:** supervision (equal), writing – review and editing (equal). **Shahrokh F. Shariat:** writing – review and editing (equal). **Takahiro Kimura:** conceptualization (equal), supervision (equal), writing – review and editing (equal). **Akihiro Hirakawa:** data curation (equal), formal analysis (equal), supervision (equal), writing – original draft (equal).

## Ethics Statement

The Certified Review Board of The Jikei University School of Medicine exempted this cohort study from review and consent, as it used anonymized, publicly available clinical trial data.

## Conflicts of Interest

Wataru Fukuokaya certifies that all conflicts of interest, including specific financial interests and relationships and affiliations relevant to the subject matter or materials discussed in the manuscript (e.g., employment/affiliation, grants or funding, consultancies, honoraria, stock ownership or options, expert testimony, royalties, or patents filed, received, or pending), are as follows: Pawel Rajwa is a consultant for Janssen. Shahrokh Shariat owns or co‐owns the following patents: Methods to determine prognosis after therapy for prostate cancer (granted September 6, 2002), Methods to determine prognosis after therapy for bladder cancer (granted June 19, 2003), Prognostic methods for patients with prostatic disease (granted August 5, 2004), and Soluble Fas: urinary marker for the detection of bladder transitional cell carcinoma (granted July 20, 2010); he has a consulting or advisory role with Astellas, AstraZeneca, Bayer, BMS, Cepheid, Ferring, Ipsen, Jansen, Lilly, MSD, Olympus, Pfizer, Pierre Fabre, Roche, Sanochemia, Sanofi, Takeda, Urogen, and Wolff. Takahiro Kimura is a paid consultant/advisor for Astellas, Bayer, Janssen, and Sanofi. Akihiro Hirakawa reports honoraria (personal) from Astellas Pharma, Ono Pharmaceutical, Novartis Pharma K.K., Kissei Pharmaceutical, Nippon Shinyaku, Chugai Pharmaceuticals, Taiho Pharmaceutical, Kyowa Kirin Co. Ltd., AbbVie, Takeda Pharmaceuticals, Janssen Pharmaceutical K.K. The other authors declare no conflicts of interest associated with this manuscript.

## Supporting information


**Appendix S1:** The Kaplan–Meier plots of the PROSELICA and the TROPIC trials. The Kaplan–Meier plots for OS and PFS of patients participating in the PROSELICA and the TROPIC trials. For the PROSELICA trial, patients receiving cabazitaxel 20 mg/m^2^ (C20) are indicated in blue, while those treated with cabazitaxel 25 mg/m^2^ (C25) are shown in red (A and C). For the TROPIC trial, patients receiving mitoxantrone are indicated in blue, and those treated with cabazitaxel 25 mg/m^2^ (C25) are shown in red (B and D). The red‐ and blue‐colored bands indicate 95% confidence intervals.
**Figure S2:** The distributions of baseline risk of poor outcomes. Histograms showing the baseline risk of poor OS and PFS, estimated by multivariable Cox proportional hazards regression models. Increased risk scores indicate increased risk of poor outcomes.
**Figure S3:** Calibration plots comparing predicted 24‐month OS and 6‐month PFS probabilities, estimated from OS and PFS risk models, to observed survival probabilities calculated from Kaplan–Meier estimates stratified by risk quartile. Bootstrapping with 500 resamples was used to estimate bias‐corrected predicted survival probabilities and 95% confidence intervals by subgroups.
**Figure S4:** The incidence of treatment‐related adverse events based on risk quartiles. The bar charts showing the incidence of grade 3 or higher non‐hematological treatment‐related adverse events (trAEs), grade 3 or higher neutropenia, and febrile neutropenia based on risk quartiles. The proportion of patients receiving cabazitaxel 25 mg/m^2^ (C25) is shown in red, and the proportion treated with 20 mg/m^2^ (C20) is shown in blue. Patients were ranked by baseline risk of poor OS or PFS and divided into equal groups. Q1 represents the lowest‐risk group, and Q4 represents the highest‐risk group.
**Figure S5:** Variable‐importance plots for OS and PFS. Variable importance was evaluated using a weighted sum of how often each variable was split in the causal survival forest model, without considering the split stage.
**Table S1:** Baseline characteristics of patients participated in the TROPIC trial.
**Table S2:** Baseline characteristics by overall survival individualized treatment effect quartile.
**Table S3:** Baseline characteristics by progression‐free survival individualized treatment effect quartile.

**Data S1:** cam471507‐sup‐0002‐Supinfo1.docx.

## Data Availability

Data are available at https://vivli.org/.
