# Supplementary material for: Heterogeneity in Treatment Effects of Reduced Versus Standard Dose of Cabazitaxel in Metastatic Castration‐Resistant Prostate Cancer
Source: Cancer Med. 2026 Jan 9;15(1):e71507. doi: 10.1002/cam4.71507 (PMC12788979; doi:10.1002/cam4.71507)
Supplement: Supplementary file 1 — Appendix S1: The Kaplan–Meier plots of the PROSELICA and the TROPIC trials. The Kaplan–Meier plots for OS and PFS of patients participating in the PROSELICA and the TROPIC trials. For the PROSELICA trial, patients receiving cabazitaxel 20 mg/m2 (C20) are indicated in blue, while those treated with cabazitaxel 25 mg/m2 (C25) are shown in red (A and C). For the TROPIC trial, patients receiving mitoxantrone are indicated in blue, and those treated with cabazitaxel 25 mg/m2 (C25) are shown in red (B and D). The red‐ and blue‐colored bands indicate 95% confidence intervals. Figure S2: The distributions of baseline risk of poor outcomes. Histograms showing the baseline risk of poor OS and PFS, estimated by multivariable Cox proportional hazards regression models. Increased risk scores indicate increased risk of poor outcomes. Figure S3: Calibration plots comparing predicted 24‐month OS and 6‐month PFS probabilities, estimated from OS and PFS risk models, to observed survival probabilities calculated from Kaplan–Meier estimates stratified by risk quartile. Bootstrapping with 500 resamples was used to estimate bias‐corrected predicted survival probabilities and 95% confidence intervals by subgroups. Figure S4: The incidence of treatment‐related adverse events based on risk quartiles. The bar charts showing the incidence of grade 3 or higher non‐hematological treatment‐related adverse events (trAEs), grade 3 or higher neutropenia, and febrile neutropenia based on risk quartiles. The proportion of patients receiving cabazitaxel 25 mg/m2 (C25) is shown in red, and the proportion treated with 20 mg/m2 (C20) is shown in blue. Patients were ranked by baseline risk of poor OS or PFS and divided into equal groups. Q1 represents the lowest‐risk group, and Q4 represents the highest‐risk group. Figure S5: Variable‐importance plots for OS and PFS. Variable importance was evaluated using a weighted sum of how often each variable was split in the causal survival forest model, without con [file CAM4-15-e71507-s002.docx]

Table of Contents

[Supplementary Figure 1 2](#_Toc190195130)

[Supplementary Figure 2 3](#_Toc190195130)

[Supplementary Figure 3 4](#_Toc190195130)

[Supplementary Figure 4 5](#_Toc190195130)

[Supplementary Figure 5 6](#_Toc190195130)

[Supplementary Table 1 7](#_Toc190195130)

[Supplementary Table 2 8](#_Toc190195130)

[Supplementary Table 3 9](#_Toc190195130)


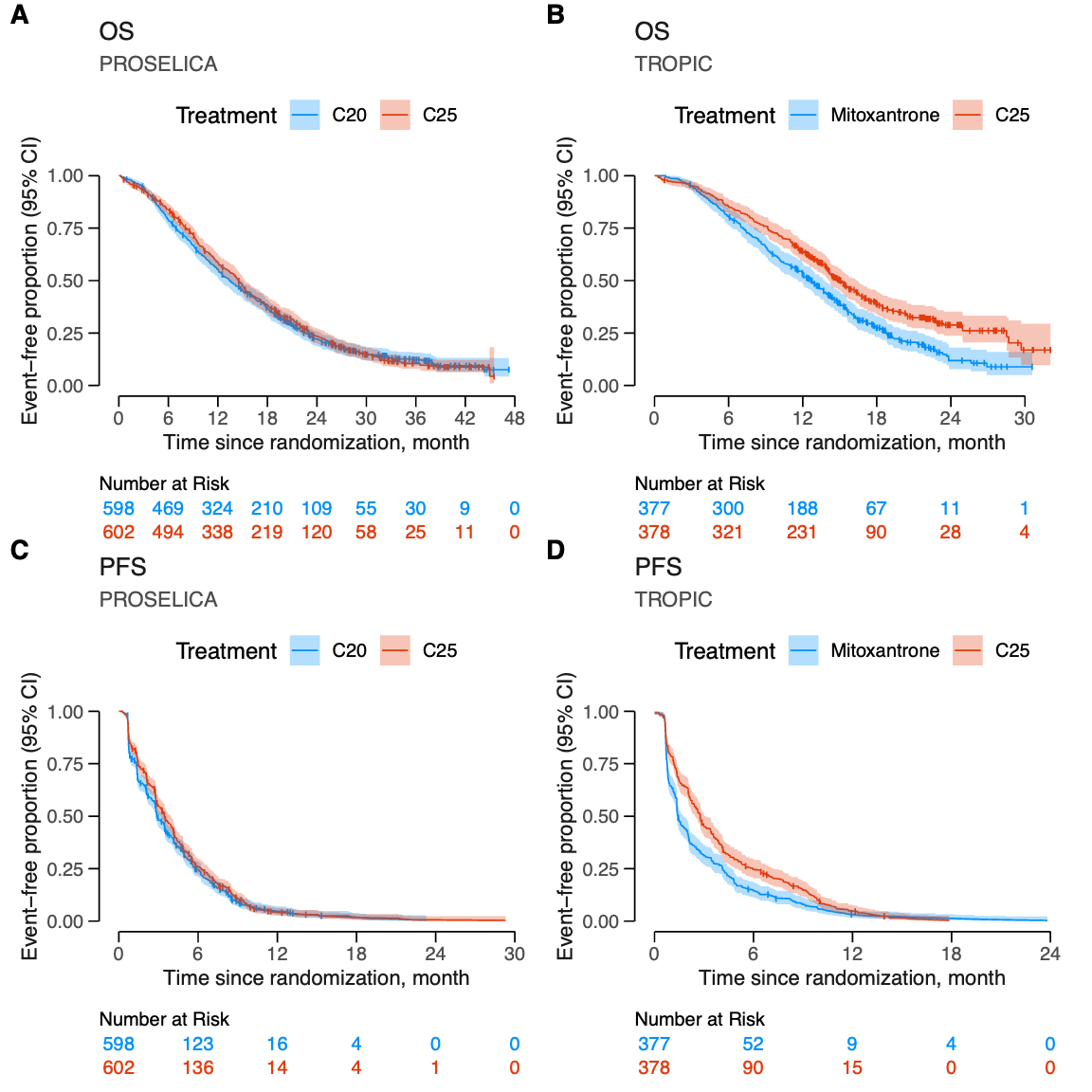


#### **Supplementary Figure 1. The Kaplan-Meier plots of the PROSELICA and the TROPIC trials**

The Kaplan-Meier plots for OS and PFS of patients participating in the PROSELICA and the TROPIC trials. For the PROSELICA trial, patients receiving cabazitaxel 20 mg/m2 (C20) are indicated in blue, while those treated with cabazitaxel 25 mg/m2 (C25) are shown in red (A and C). For the TROPIC trial, patients receiving mitoxantrone are indicated in blue, and those treated with cabazitaxel 25 mg/m2 (C25) are shown in red (B and D). The red- and blue-colored bands indicate 95% confidence intervals.


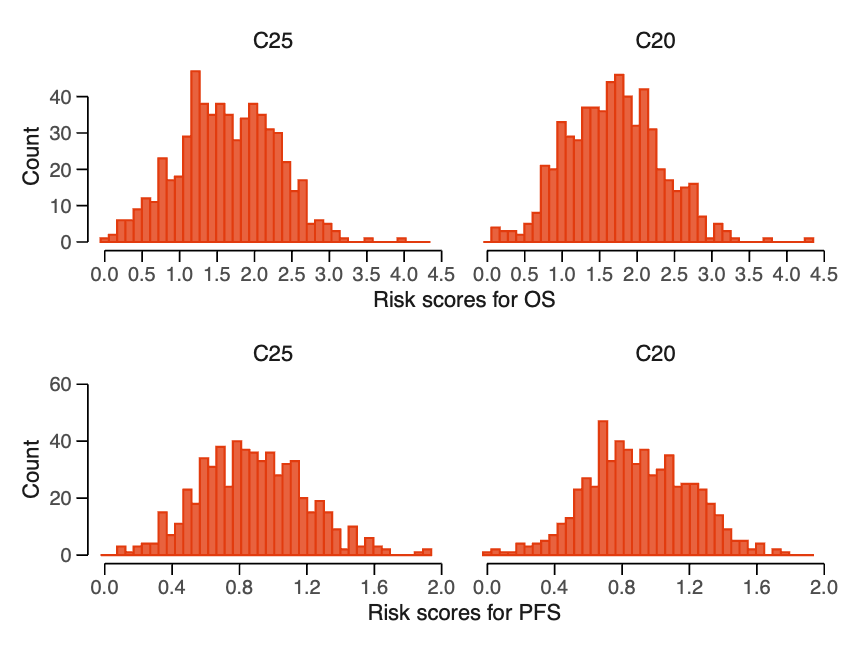


#### **Supplementary Figure 2. The distributions of baseline risk of poor outcomes**

Histograms showing the baseline risk of poor OS and PFS, estimated by multivariable Cox proportional hazards regression models. Increased risk scores indicate increased risk of poor outcomes.


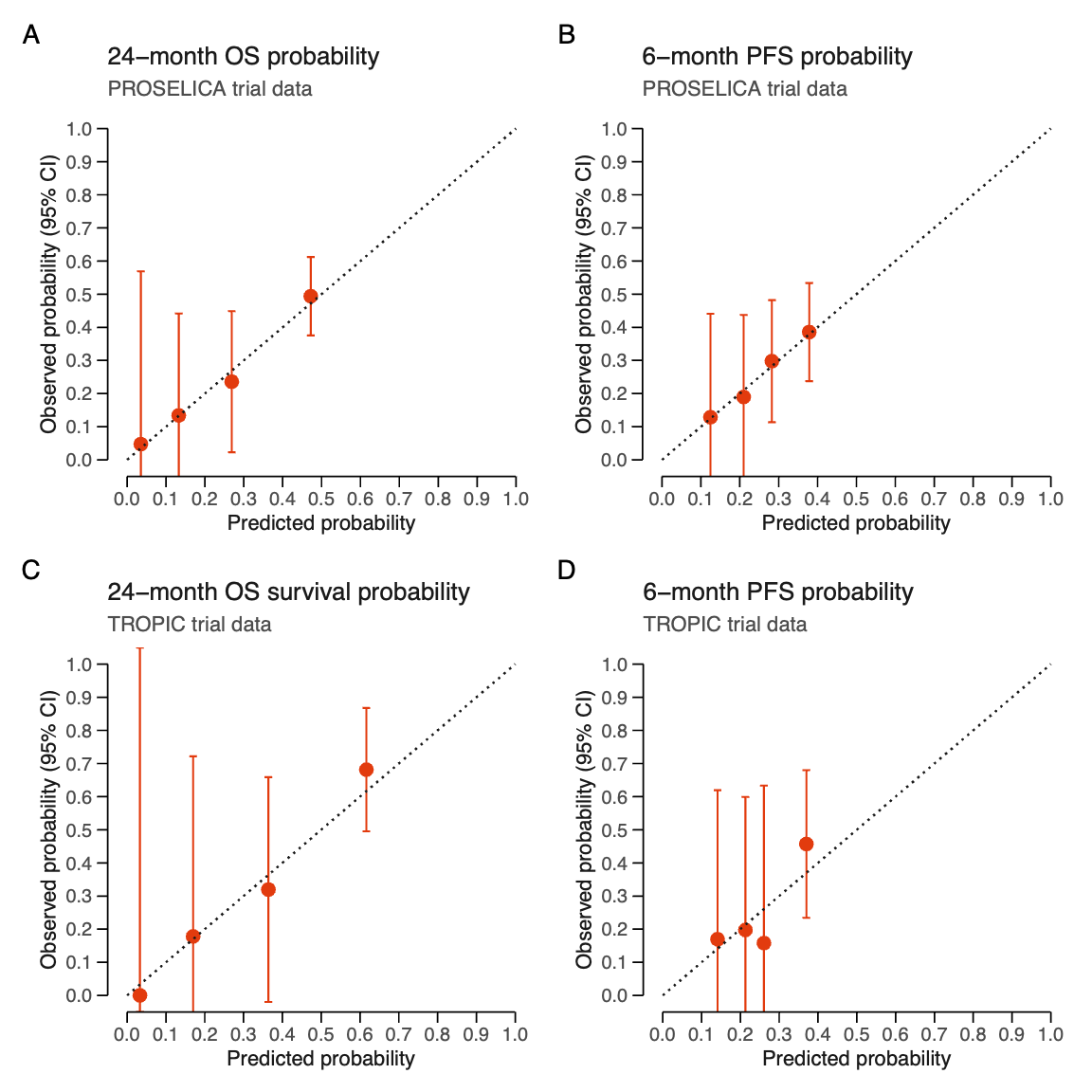


#### **Supplementary Figure 3. Calibration plots**

Calibration plots comparing predicted 24-month OS and 6-month PFS probabilities, estimated from OS and PFS risk models, to observed survival probabilities calculated from Kaplan-Meier estimates stratified by risk quartile. Bootstrapping with 500 resamples was used to estimate bias-corrected predicted survival probabilities and 95% confidence intervals by subgroups.


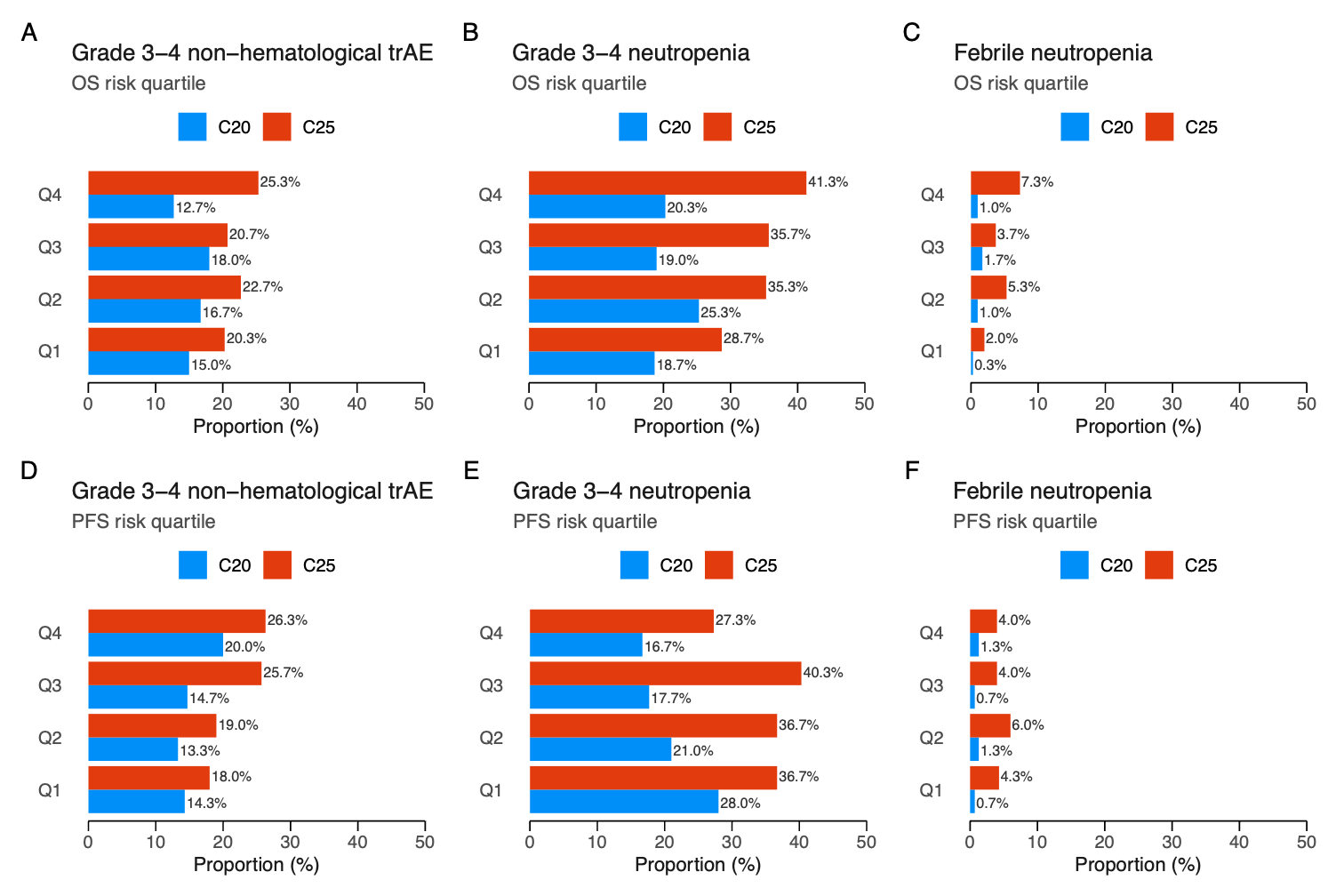


#### **Supplementary Figure 4. The incidence of treatment-related adverse events based on risk quartiles**

The bar charts showing the incidence of grade 3 or higher non-hematological treatment-related adverse events (trAEs), grade 3 or higher neutropenia, and febrile neutropenia based on risk quartiles. The proportion of patients receiving cabazitaxel 25 mg/m2 (C25) is shown in red, and the proportion treated with 20 mg/m2 (C20) is shown in blue. Patients were ranked by baseline risk of poor OS or PFS and divided into equal groups. Q1 represents the lowest-risk group, and Q4 represents the highest-risk group.


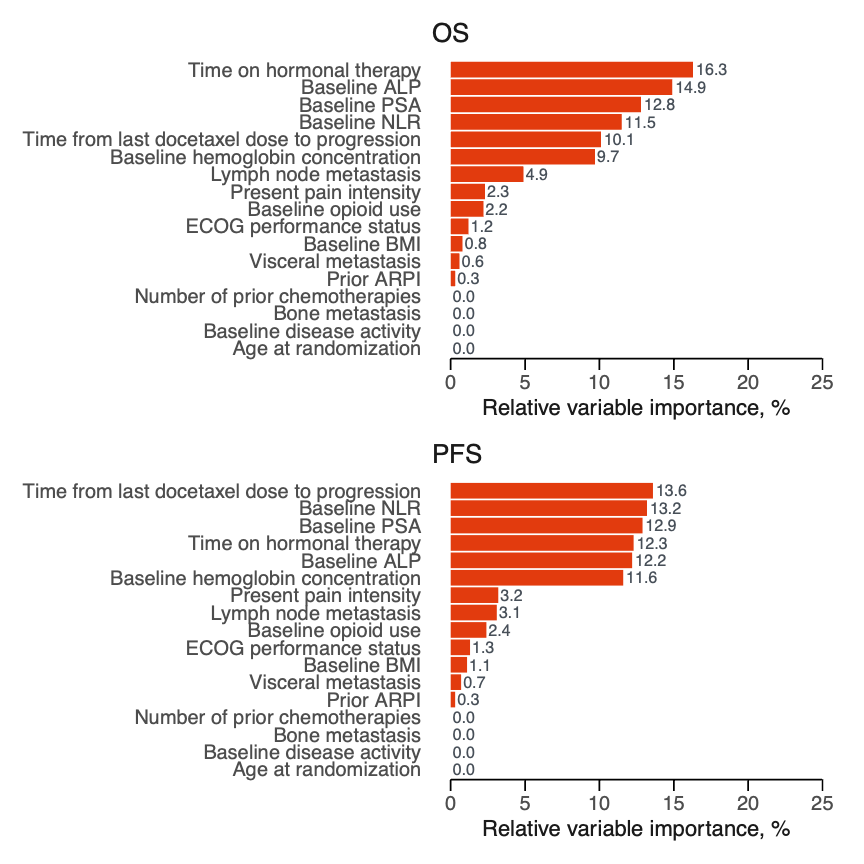


#### **Supplementary Figure 5. Variable importance plots**

Variable-importance plots for OS and PFS. Variable importance was evaluated using a weighted sum of how often each variable was split in the causal survival forest model, without considering the split stage.

| **Supplementary Table 1. Baseline characteristics of patients participated in the TROPIC trial** | |
| --- | --- |
|  | **C25** |
| **Characteristic** | **N = 378** |
| Age at baseline, n (%) |  |
| 60 to 80 | 73 (19.3) |
| More than 80 | 305 (80.7) |
| Body mass index, n (%) |  |
| Underweight to normal weight | 110 (29.6) |
| Pre-obesity | 166 (44.7) |
| Obesity class I or more | 95 (25.6) |
| Missing | 7 |
| ECOG performance status, n (%) |  |
| 0 | 136 (36.7) |
| 1 or more | 235 (63.3) |
| Missing | 7 |
| Present pain intensity, Median (IQR) | 2.0 (0.0 to 3.0) |
| Missing | 44 |
| Baseline opioid use, n (%) | 113 (29.9) |
| Prior androgen receptor pathway inhibitor, n (%) | 0 (0.0) |
| Baseline disease activity, n (%) |  |
| Increasing PSA at baseline | 302 (79.9) |
| Stable or declining PSA at baseline | 76 (20.1) |
| Time from last docetaxel dose to progression, Median (IQR) | 0.8 (0.0 to 3.4) |
| Missing | 56 |
| Number of prior chemotherapies, n (%) |  |
| 1 | 260 (68.8) |
| 2 or more | 118 (31.2) |
| Time on hormonal therapy, Median (IQR) | 4.2 (2.5 to 6.6) |
| Missing | 3 |
| Baseline bone metastasis, n (%) |  |
| Abscent | 75 (19.8) |
| Present | 303 (80.2) |
| Baseline lymph node metastasis, n (%) |  |
| Abscent | 208 (55.0) |
| Present | 170 (45.0) |
| Baseline visceral metastasis, n (%) |  |
| Abscent | 277 (73.3) |
| Present | 101 (26.7) |
| Baseline hemoglobin concentration, Median (IQR) | 119.2 (109.7 to 130.0) |
| Missing | 7 |
| Baseline NLR, Median (IQR) | 3.3 (2.2 to 5.1) |
| Missing | 7 |
| Baseline ALP, Median (IQR) | 139.0 (81.0 to 277.5) |
| Missing | 9 |
| Baseline PSA, Median (IQR) | 143.9 (51.1 to 416.0) |
| Missing | 7 |
| C25, cabazitaxel 25 mg/m^2^; ECOG; eastern cooperative oncology group; | |
| PSA, prostate-specific antigen; NLR, neutrophil-to-lymphocyte ratio; ALP, alkaline phosphatase; | |
| IQR; interquartile range |  |

| **Supplementary Table 2. Baseline characteristics by overall survival individualized treatment effect quartile** | | |
| --- | --- | --- |
|  | **Q1** | **Q4** |
| **Characteristic** | **N = 300** | **N = 300** |
| Age at baseline |  |  |
| Less than 60 | 62 (20.7) | 36 (12.0) |
| 60 to 80 | 227 (75.7) | 247 (82.3) |
| More than 80 | 11 (3.7) | 17 (5.7) |
| Body mass index |  |  |
| Underweight to normal weight | 80 (26.7) | 90 (30.0) |
| Pre-obesity | 158 (52.7) | 124 (41.3) |
| Obesity class I or more | 62 (20.7) | 86 (28.7) |
| ECOG performance status |  |  |
| 0 | 99 (33.0) | 91 (30.3) |
| 1 or more | 201 (67.0) | 209 (69.7) |
| Present pain intensity | 1.0 (0.0 to 2.0) | 2.0 (1.0 to 2.0) |
| Missing | 24 | 32 |
| Baseline opioid use | 129 (43.0) | 129 (43.0) |
| Prior androgen receptor pathway inhibitor | 59 (19.7) | 99 (33.0) |
| Baseline disease activity |  |  |
| Increasing PSA at baseline | 257 (85.7) | 264 (88.0) |
| Stable or declining PSA at baseline | 43 (14.3) | 36 (12.0) |
| Time from last docetaxel dose to progression | 1.0 (0.3 to 3.0) | 1.2 (0.0 to 4.2) |
| Missing | 28 | 29 |
| Number of prior chemotherapies |  |  |
| 1 | 259 (87.2) | 228 (77.0) |
| 2 or more | 38 (12.8) | 68 (23.0) |
| Missing | 3 | 4 |
| Time on hormonal therapy | 1.7 (1.2 to 2.1) | 5.3 (4.1 to 7.6) |
| Missing | 36 | 32 |
| Baseline bone metastasis |  |  |
| Abscent | 16 (5.3) | 11 (3.7) |
| Present | 284 (94.7) | 289 (96.3) |
| Baseline lymph node metastasis |  |  |
| Abscent | 185 (61.7) | 109 (36.3) |
| Present | 115 (38.3) | 191 (63.7) |
| Baseline visceral metastasis |  |  |
| Abscent | 225 (75.0) | 213 (71.0) |
| Present | 75 (25.0) | 87 (29.0) |
| Baseline hemoglobin concentration | 121.8 (110.0 to 132.0) | 117.0 (107.0 to 126.0) |
| Baseline NLR | 3.4 (2.1 to 5.5) | 3.4 (2.5 to 5.4) |
| Missing | 2 | 3 |
| Baseline ALP | 123.5 (80.4 to 183.0) | 349.0 (176.5 to 600.0) |
| Missing | 2 | 0 |
| Baseline PSA | 148.4 (54.8 to 332.9) | 249.9 (68.3 to 687.4) |
| Missing | 3 | 2 |
|  |  |  |

| **Supplementary Table 3. Baseline characteristics by progression-free survival individualized treatment effect quartile** | | |
| --- | --- | --- |
|  | **Q1** | **Q4** |
| **Characteristic** | **N = 300** | **N = 300** |
| Age at baseline |  |  |
| Less than 60 | 47 (15.7) | 45 (15.0) |
| 60 to 80 | 236 (78.7) | 242 (80.7) |
| More than 80 | 17 (5.7) | 13 (4.3) |
| Body mass index |  |  |
| Underweight to normal weight | 99 (33.0) | 82 (27.3) |
| Pre-obesity | 134 (44.7) | 133 (44.3) |
| Obesity class I or more | 67 (22.3) | 85 (28.3) |
| ECOG performance status |  |  |
| 0 | 91 (30.3) | 103 (34.3) |
| 1 or more | 209 (69.7) | 197 (65.7) |
| Present pain intensity | 2.0 (1.0 to 2.0) | 1.0 (0.0 to 2.0) |
| Missing | 24 | 20 |
| Baseline opioid use | 139 (46.3) | 98 (32.7) |
| Prior androgen receptor pathway inhibitor | 81 (27.0) | 81 (27.0) |
| Baseline disease activity |  |  |
| Increasing PSA at baseline | 256 (85.3) | 272 (90.7) |
| Stable or declining PSA at baseline | 44 (14.7) | 28 (9.3) |
| Time from last docetaxel dose to progression | 3.2 (1.5 to 5.7) | 0.0 (0.0 to 0.7) |
| Missing | 26 | 16 |
| Number of prior chemotherapies |  |  |
| 1 | 255 (85.6) | 249 (85.0) |
| 2 or more | 43 (14.4) | 44 (15.0) |
| Missing | 2 | 7 |
| Time on hormonal therapy | 3.4 (2.1 to 5.1) | 3.2 (2.0 to 5.3) |
| Missing | 49 | 43 |
| Baseline bone metastasis |  |  |
| Abscent | 25 (8.3) | 18 (6.0) |
| Present | 275 (91.7) | 282 (94.0) |
| Baseline lymph node metastasis |  |  |
| Abscent | 156 (52.0) | 136 (45.3) |
| Present | 144 (48.0) | 164 (54.7) |
| Baseline visceral metastasis |  |  |
| Abscent | 216 (72.0) | 216 (72.0) |
| Present | 84 (28.0) | 84 (28.0) |
| Baseline hemoglobin concentration | 113.5 (105.0 to 122.0) | 125.0 (118.0 to 131.0) |
| Baseline NLR | 2.8 (2.1 to 4.6) | 3.8 (2.5 to 5.8) |
| Missing | 1 | 3 |
| Baseline ALP | 132.5 (78.5 to 240.0) | 218.0 (107.0 to 395.0) |
| Missing | 0 | 1 |
| Baseline PSA | 82.2 (29.3 to 309.8) | 233.9 (94.4 to 477.0) |
| Missing | 2 | 2 |
