# Supplementary material for: Heterogeneity in Treatment Effects of Reduced Versus Standard Dose of Cabazitaxel in Metastatic Castration‐Resistant Prostate Cancer
Source: Cancer Med. 2026 Jan 9;15(1):e71507. doi: 10.1002/cam4.71507 (PMC12788979; doi:10.1002/cam4.71507)
Supplement: Supplementary file 2 — Data S1: cam471507‐sup‐0002‐Supinfo1.docx. [file CAM4-15-e71507-s001.docx]

### Supplementary Methods

#### Risk-modeling approach for heterogeneity in treatment effect

Following the method of previous research [1,2], we evaluated the treatment effect of cabazitaxel 25 mg/m^2^ (C25) versus 20 mg/m^2^ (C20) within risk groups estimated using an internally derived risk prediction model in the PROSELICA dataset. We pre-defined a set of 18 candidate variables for risk prediction models, including age (continuous), Eastern Cooperative Oncology Group performance status (0 versus ≥1), body mass index (continuous), bone metastasis at baseline (absent versus present), lymph node metastasis at baseline (absent versus present), visceral metastasis at baseline (absent versus present), baseline prostate-specific antigen levels (PSA) (continuous), disease activity at baseline (stable or declining PSA versus increasing PSA), time from last docetaxel dose to progression (continuous), prior use of androgen receptor pathway inhibitor (no versus yes), number of prior chemotherapy regimens (1 versus ≥2), time from androgen deprivation therapy (continuous), baseline pain (measured by mean present pain intensity or mean analgesic score), prior use of analgesics including opioids (no versus yes), baseline hemoglobin (continuous), baseline neutrophil-to-lymphocyte ratio (continuous), baseline alkaline phosphatase (continuous), and baseline lactate dehydrogenase (continuous). Because the TROPIC trial did not measure lactate dehydrogenase, we conducted the risk-modeling process using the 17 remaining variables. Additionally, we analyzed age and body mass index as categorical variables, as these variables were categorized during the data anonymization process.

We applied the following risk-modeling process to overall survival (OS) and progression-free survival (PFS) as per the trial protocol [3].

1. The set of 17 candidate variable for risk model was imputed using the multivariable imputation by chained equations (MICE) algorithm [4]. Fifty imputed datasets were generated with MICE, and the variable selection process (Step 2) was performed on each dataset. Fifty chained imputation iterations were performed to ensure convergence in each imputation.
2. For each imputed dataset, we fit all 2^17^ possible variable combinations with a Cox proportional hazards regression model and calculated the Akaike Information Criteria (AIC) for all models. To estimate a baseline risk score for each patient independent of their treatment assignment, we first developed a multivariable Cox proportional hazards regression model that included the selected baseline covariates and a term for the randomized treatment assignment (C25 vs. C20). Following the approach described by Pocock et al.[5], we then calculated the risk score for each patient from this model's coefficients by setting the treatment assignment term to the value corresponding to the control arm (C20) for all patients. Per imputed dataset, we selected the model with the combination of risk variables that produced the lowest AIC value for the candidate risk model. Due to the large number of possible models, we did not include interactions between variables in the candidate risk models.
3. To combine the results across all imputed datasets, we selected the variables for the final risk model from the model most frequently selected across all 50 imputed datasets.
4. After selecting the risk model variables, we computed risk scores for each outcome using the Cox proportional hazards regression model based on the intention-to-treat population of the PROSELICA trial. We calculated risk scores as the linear combination of the log hazard ratio of each variable and patient characteristics. For interpretability, we scaled risk scores so that the lowest risk score was 0 and higher risk scores corresponded with increased probability of events. We assessed the discriminatory ability of the risk model with the Harrell’s c-statistic [6]. We evaluated model calibration visually using calibration plots. This analysis used bootstrapping with 500 resamples to obtain bias-corrected estimates of predicted 24-month OS and 6-month PFS probabilities. We externally validated the final risk model using patient-level data from individuals treated with C25 in the TROPIC trial.
5. We grouped participants based on quartiles of risk scores calculated in Step 4. To provide clinically meaningful measures for absolute treatment effect, we estimated the treatment effect of C25 versus C20 for each group with the difference in restricted mean survival time (RMST). We defined the truncation times as 24 months for OS and 6 months for PFS.

#### Effect-modeling approach of heterogeneity in treatment effect

This analysis modeled treatment effects directly from the data. Similar to the risk-based modeling approach, this analysis involved fitting an effect-based model and evaluating treatment effects across patients grouped by model predictions. We relied on repeated data-splitting methodologies to mitigate overfitting bias and for making inferences. We followed the generic machine learning analysis framework proposed by Chernozhukov et al. for this analysis [7]. The procedure is illustrated below.

1. In Step A, we randomly split the overall PROSELICA dataset 1:1 into two halves: auxiliary and main sets.
2. In Step B, we performed imputation modeling and effect modeling using the auxiliary set. We generated 20 imputed datasets with the auxiliary set using the MICE algorithm with 50 iterations. We constructed imputation models using only the auxiliary set to maintain separation between auxiliary and main sets. We trained a causal survival forest algorithm [8], a data-driven non-parametric method to identify factors leading to HTE, for each imputed dataset. We followed the previous study using the causal survival forest algorithm [9]. We chose the same 17 covariates used in the risk-modeling approach. We tuned the following hyperparameters for the causal survival forest algorithm with 10000 trees during training for each imputed dataset: sample fraction, honest fraction, and the number of variables considered for each split.
3. In Step C, we imputed the main set 20 times with the imputation model used for the auxiliary set, and estimated predicted individualized treatment effects (ITEs) with the imputed main set and causal survival forest model trained with the corresponding auxiliary set. We assigned a final predicted ITE to each participant as the average of ITE across 20 imputed datasets. We ranked participants with predicted ITEs and grouped them by quartiles. Within each ITE group, we calculated observed differences in RMST and their 95% confidence intervals for each outcome.
4. We repeated Steps A to C 100 times and aggregated the results of each iteration in Step D. We estimated the observed difference in RMST in each quartile as the median of estimated difference in RMST across the 100 iterations, and calculated 90% confidence intervals as the medians of the one hundred 95% confidence intervals.
5. We calculated variable importance based on the causal survival forest algorithm for OS and PFS to assess the degree to which each covariate contributed to treatment effect heterogeneity. Additionally, we compared baseline characteristics according to predicted ITE quartiles.

#### References

[1] Kent DM, Rothwell PM, Ioannidis JPA, Altman DG, Hayward RA. Assessing and reporting heterogeneity in treatment effects in clinical trials: a proposal. Trials 2010;11:85.

[2] Rekkas A, Paulus JK, Raman G, Wong JB, Steyerberg EW, Rijnbeek PR, et al. Predictive approaches to heterogeneous treatment effects: a scoping review. BMC Med Res Methodol 2020;20:264.

[3] Eisenberger M, Hardy-Bessard A-C, Kim CS, Géczi L, Ford D, Mourey L, et al. Phase III study comparing a reduced dose of cabazitaxel (20 mg/m2) and the currently approved dose (25 mg/m2) in postdocetaxel patients with metastatic castration-resistant prostate cancer-PROSELICA. J Clin Oncol 2017;35:3198–206.

[4] van Buuren S, Groothuis-Oudshoorn K. mice: Multivariate Imputation by Chained Equations inR. J Stat Softw 2011;45. https://doi.org/10.18637/jss.v045.i03.

[5] Pocock SJ, Ariti CA, Collier TJ, Wang D. The win ratio: a new approach to the analysis of composite endpoints in clinical trials based on clinical priorities. Eur Heart J 2012;33:176–82.

[6] Harrell FE Jr, Califf RM, Pryor DB, Lee KL, Rosati RA. Evaluating the yield of medical tests. JAMA 1982;247:2543–6.

[7] Chernozhukov V, Demirer M, Duflo E, Fernández-Val I. Generic machine learning inference on heterogeneous treatment effects in randomized experiments, with an application to immunization in India. Cambridge, MA: National Bureau of Economic Research; 2018. https://doi.org/10.3386/w24678.

[8] Cui Y, Kosorok MR, Sverdrup E, Wager S, Zhu R. Estimating heterogeneous treatment effects with right-censored data via causal survival forests. ArXiv [StatME] 2020.

[9] Desai RJ, Glynn RJ, Solomon SD, Claggett B, Wang SV, Vaduganathan M. Individualized treatment effect prediction with machine learning - salient considerations. NEJM Evid 2024;3:EVIDoa2300041.
